# Effects of Attenuated *S. agalactiae* Strain YM001 on Intestinal Microbiota of Tilapia Are Recoverable

**DOI:** 10.3389/fmicb.2018.03251

**Published:** 2019-01-09

**Authors:** Ming Li, Liping Li, Ting Huang, Yu Liu, Aiying Lei, Chunxia Ma, Fuyan Chen, Ming Chen

**Affiliations:** ^1^Guangxi Key Laboratory of Aquatic Genetic Breeding and Healthy Aquaculture, Guangxi Institute of Fisheries, Nanning, China; ^2^Guangxi Key Laboratory of Animal Vaccines and Diagnostics, Department of Bacteriology, Guangxi Veterinary Research Institute, Nanning, China

**Keywords:** gut microbiota, oral vaccine, *Streptococcus agalactiae*, tilapia, microbiology

## Abstract

Previously, we constructed and characterized the vaccine efficacy of attenuated *S. agalactiae* strain YM001 in tilapia. In this study, the potential impacts of YM001 on the tilapia intestinal microbiota were assessed by qPCR and 16S rRNA sequencing methods. The results showed that YM001 distributed unevenly in different parts of intestine, peaked in the intestine at 12 h after oral administration, and then declined gradually. YM001 caused 0% mortality of fish during the entire experimental period, while the referent strain HN016 caused 100% mortality at 3 d after oral administration. However, the intestinal microbiota could be changed by YM001, the diversity of intestinal microbiota decreased first and gradually recovered after oral administration. The diversity of intestinal microbiota of tilapia was negatively correlated with the content of HN016 in the intestinal tract. The oral YM001 mainly changed the abundance of Streptococcus, Cetobacterium, Akkermansia, Romboutsia, Bacteroides, Brevinema, Lachnospiraceae_NK4A136-group, coprothermobactter, presiomonas, and Roseburia in intestine. The present study indicate that oral administration of YM001 altered the diversity and composition of intestinal microbiota in tilapia, but these change were only temporary, non-lethal, and recoverable. The results provide a more comprehensive experimental basis for the safety of oral YM001 vaccines.

## Introduction

*Streptococcus agalactiae*, or Group B streptococcus (GBS) is an important pathogen causing sepsis and meningitis in newborns and mastitis in bovine (Edwards and Baker, 2005; Tazi et al., 2011; Edmond et al., 2012; Lamagni et al., 2013). *S. agalactiae* is also a common aquaculture pathogen that can harm different fish species, such as tilapia, Barcoo grunter (*Scortum barcoo*), golden pompano (*Trachinotus blochii*), ya-fish (*Schizothorax potanini*), giant queensland grouper (*Epinephelus lanceolatus*), and silver pomfret (*Pampus argenteus*) (Jafar et al., 2008; Amal et al., 2012; Li et al., 2015). Tilapia is an important aquaculture species. Outbreaks of *S. agalactiae* in tilapia have cost more than 40 million dollars every year worldwide (Mian et al., 2009; Chen et al., 2012). Currently, *S. agalactiae* control in tilapia mainly relies on antibiotics, but the overuse of antibiotics will lead to food safety and problems with drug-resistant strains. Therefore, the use of antibiotics in aquaculture is severely restricted (Depaola et al., 1995; Baquero et al., 2008).

Vaccines are the safest and most reliable method for the prevention of human and animal infectious diseases, but most of them are given by injection. However, in the field of aquatic products, the use of injections for immunization is greatly limited due to the large population of aquatic animals and the greater stress response resulted from the injection of vaccines. Compared to injectable vaccines, oral vaccines are more suitable for aquaculture animals. These vaccines are generally classified as live attenuated vaccines (LAVs), inactivated vaccines and subunit vaccines. Compared with inactivated vaccines or subunit vaccines, oral LAV have great advantages by avoiding the degradation of intestinal enzymes, directly entering the liver, kidney, spleen, and other immune related sites and simultaneously stimulating systemic immunity and local mucosal immunity (Chen et al., 2015; Makesh et al., 2015). Because of the above advantages, three types of LAVs against *S. agalactiae* in tilapia have been developed recently (Pridgeon and Klesius, 2013; Huang et al., 2014; Li et al., 2015).

Although oral LAVs have many advantages in application, their effects on the intestinal microbiota cannot be ignored. Long-term natural selection and differences in habitat, diet and habits have led to the formation of unique microbial symbiosis among different animals (Rawls et al., 2004; Ley et al., 2008). For fish, the micro-ecosystem composed of gut microorganisms is also formed during long-term historical evolution. These microorganisms are involved in the development of fish epithelial barrier, nutrition, digestion, as well as immunity (Gómez and Balcazar, 2008; Kelly, 2010; Roeselers et al., 2011; Sommer and Backhed, 2013; Xia et al., 2014). However, the balance of intestinal community composition could be altered by many factors (Xia et al., 2014), including stress (O'mahony et al., 2009; Kelly, 2010), antibiotic exposure (Tanaka et al., 2009), nutritional status (Turnbaugh et al., 2009), age (Hopkins et al., 2002), degree of hygiene (Schmidt et al., 2011), and bacterial infection (Dethlefsen et al., 2006). Gut microorganisms are continuously shaping the development of their host's immune system, directly modulating the innate and adaptive immune responses (Sommer and Backhed, 2013; Barroso-Batista et al., 2015). Differences in the host's intestinal microbiota can affect the effectiveness of the vaccine. For instance, intestinal microbiome composition correlates significantly with the immunogenicity of rotavirus vaccine (RVV) and may contribute to the diminished RVV immunogenicity. RVV response is correlated with an increased abundance of *Streptococcus bovis* and a decreased abundance of *Bacteroidetes phylum* (Harris et al., 2017). Therefore, it is necessary to analyze the effect of oral vaccines on intestinal microbiota.

In the previous study, we attenuated and domesticated the virulent *S. agalactiae* strain HN016 and obtained a safe, stable, and highly immunogenic attenuated *S. agalactiae* strain YM001. Oral immunization of tilapia with this strain produced a good immune protection (Chen et al., 2012; Li et al., 2015; Wang et al., 2015). Despite this, the effect of oral administration of YM001 on tilapia intestinal microbiota is still unknown. In this study, we analyzed the pathogenicity of oral administration of YM001 as well as its distribution in the intestine and influence on intestinal microbiota in tilapia using quantitative PCR and 16S bacterial sequencing with the hope to provide a more comprehensive experimental basis for the safety of YM001 oral vaccines.

## Materials and Methods

### Ethics Statement

This study was carried out in accordance with the principles of good animal practice as defined by the European Union guidelines for the handling of laboratory animals (http://ec.europa.eu/environment/chemicals/lab_animals/home_en.htm) and the protocol was approved by Guangxi Institute of Fisheries Animal Ethics Committee and Guangxi Medical University Animal Ethics Committee. The institute did not issue a number or ID to this animal study, because the studied fish are not an endangered or protected species.

### Bacterial Strains and Fish

*S. agalactiae* strain HN016 (serotype Ia) was isolated from an outbreak epidemical disease in tilapia from Hainan, China in 2010 (Chen et al., 2012). *S. agalactiae* strain YM001 was obtained by continuously passaging HN016 *in vitro* for 840 passages (Li et al., 2015). These two strains were stored at −80°C. Non-infected *Nile tilapia* was obtained from the National Tilapia Seed Farm (Nanning, Guangxi, China), with average weight of 155.45 ± 20.85 g. Prior to experiments, the fishes were acclimated in plastic tanks (800 L each) with a stocking rate of 4 g/L at 30 ± 4°C for 2 weeks. The experimental fishes were checked randomly to verify pathogen free by bacteriological analysis of the brain and kidney samples.

### Oral Administration

The bacteria for oral gavage were recovered and cultured as described previously (Li et al., 2015). In brief, the stored HN016 and YM001 were thawed, streaked onto 5.0% sheep blood agar plates, and incubated at incubator (Jing Hong, China) 28°C for 24 h. Then single colony of every strain was inoculated in sterile tryptone soy broth (TSB) and incubated at 28°C for 24 h under low agitation. The concentration of the bacteria was determined as colony forming unit (CFU) per mL by plating 100 μL of 10-fold serial dilutions onto blood agar plates. The details are as follow: 100 μL culture of HN016 or YM001was added to 900 μL PBS and blended. Then 100 μL diluent was added to another 900 μL PBS and blended. By the same method, the culture was 10-fold serial diluted to different concentration. Hundred micro liter diluent with different concentration were plated onto the blood agar plates and then incubated at 28°C for 24 h. Only the agar plates with 30–100 colony of bacteria was selected to calculated the concentration of primary culture.

A total of 105 fishes were orally administered with HN016 or YM001 at the dose of 1.0 × 10^9^ CFU/fish (35 fishes/tank, with 3 replicates), and the control group was treated with 1.0 mL/fish of TSB. The experimental fishes were cultured in plastic tanks (800 L each) at 30 ± 4°C, then monitored and fed twice a day for 30 days. Five fishes in each group were sampled randomly at 0h, 12h, 24h, 3d, 7d, 15d after oral gavage. Sampled fishes were dissected immediately with sterile scissors. The intestine was aseptically removed from their abdominal cavity, and cut into duodenum, foregut, midgut and hindgut, separately. All the samples then were stored at −80°C immediately until use.

### DNA Extraction and Real-Time RT-PCR Assays

Microbial DNA was extracted from fish intestine samples by using the Bacterial DNA Kit (50) (Takara, Japan) following the manufacturer's protocol. For each sample, DNA was extracted in triplicate to avoid bias, and the extracts from the same sample were pooled. The purity of DNA extracts was verified by electrophoresis on ethidium bromide staining 1% agarose gels, and their concentration was analyzed spectrophotometrically. To detect the distribution of YM001 or HN016 in tilapia intestines, specific primers and probe for *S. agalactiae* were design according to the cfb gene (Gene ID: 3686873). The Primer and probe sequences are listed in Table 1. Real-time PCR assays were performed using the Premix Ex Taq according to the protocol of Probe qPCR Kit (Takara, Japan) in a LightCycler 480 System (Roche, Germany). The PCR assays were performed at a volume of 20.0 μL comprised of 10.0 μL of SYBR Green I Master Mix (Takara, Japan), 0.5 μL of cfb-F, 0.5 μL of cfb-R, 0.5 μL of TaqMan Probe, 4.0 μL of template DNA and 7.5 μL of ddH_2_O. The cycling parameters were 30 s at 95°C followed by 40 cycles of 5 s at 95°C 5 and 30 s at 60°C. In blank control group, ddH_2_O was used to replace the template DNA. All Real-time PCR reactions were performed in triplicate.

**Table 1:** Sequences of primers and probe.

**Primer**	**Sequence (5^**′**^ to 3^**′**^)**
cfb-F	CGGTTAATGAGGCTATTACTAGTG
cfb-R	ATCTGTTAAGGCTTCTACACGAC
cfb-P probe	FAM-TTCATTGCGTGCCAACCCTGAGACA-Eclipse

### Illumina HiSeq Sequencing

The extracted genomic DNA samples were diluted to 1 ng/μl with sterile water and used as templates to amplify the selected sequencing region using specific primers with barcodes and DNA polymerase with high efficiency and high fidelity. The PCR products were separated on 2% agarose gels, purified and prepared as libraries using TruSeq® DNA PCR Free Sample Preparation Kit. The constructed libraries were analyzed using Tapestation for quality control and Qubit and qPCR for quantification and sequenced using a Hiseq 2500PE250 platform.

The obtained FASTQ data were demultiplexed. After removal of barcodes and poor quality data, the obtained effective reads were analyzed using Upplese software (Uparse v7.0.1001, http://drive5.com/uparse/) and clustered into operational taxonomic units (OTUs) based on 97% identity. The sequences of each OUT with the highest abundance were selected as the representative sequence and used for annotation against the GreenGene database (http://greengenes.secondgenome.com) using the RDP Classifier (Version 2.2, http://sourceforge.net/projects/rdp-classifier/) at the setting threshold of 0.8–1 to calculate the community composition of each sample at the levels of kingdom, phylum, class, order, family, genus, and species.

The data for each sample was normalized and subjected to Alpha diversity analysis and Beta diversity analysis.

## Results

### Disease Symptoms of the Experimental Fish

All fish in the YM001 group showed no significant changes except that their appetite dropped at 24–72 h after oral administration of YM001. The appetite returned to normal at 4 d after the administration. No fish in the YM001 group died during the entire experiment period. By contrast, fish in the HN016 group showed typical symptoms of *S. agalactiae* infection at 24 h after administration of HN016 including lack of energy, abnormal swimming posture and body blackness and died 3 d after administration of HN016.

### Distribution of YM001 in Fish Intestine

**Figure 1:**
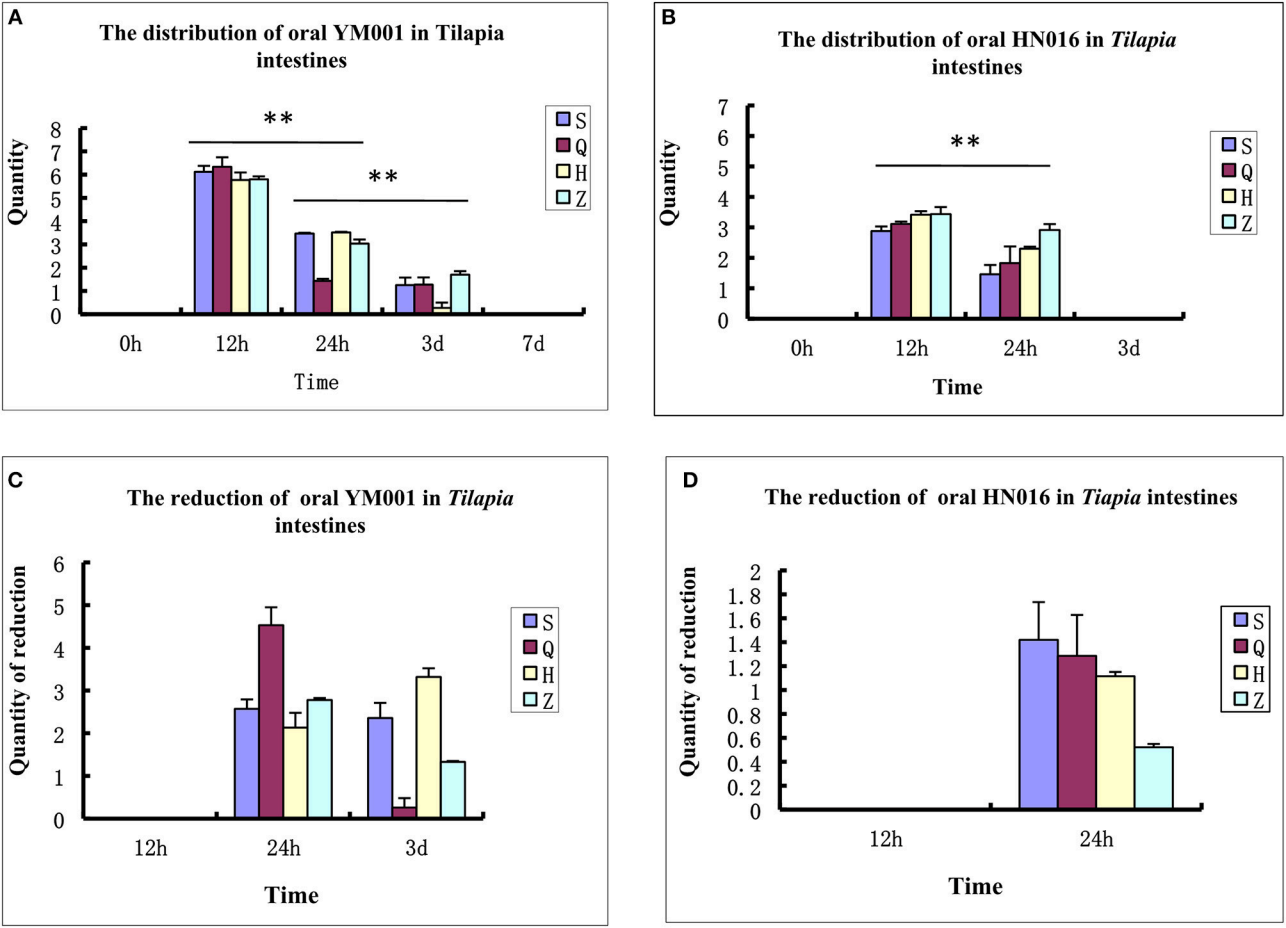
The retention time of YM001 and HN016 is different in the intestinal tract. **(A)** The distribution of YM001 in Tilapia intestines. **(B)** The distribution of HN016 in Tilapia intestines. **(C)** The reduction of YM001 in Tilapia intestines. **(D)** The reduction of HN016 in Tilapia intestines. Real-time RT-PCR was performed in triplicate for each sample. The data were provided as the mean fold changes (means ± S.D., *n* = 3) relative to the control group. The statistical significance was calculated by the Student's *t*-test (^**^p < 0.01).

Tilapia intestinal samples were collected at 0 h, 12 h, 24 h, 3 d, 7 d, and 15 d after oral administration of *S. agalactiae*, respectively. Their DNA were extracted and analyzed using fluorescence quantitative PCR to detect the distribution and colonization of YM001 and HN016 in the intestine of tilapia. The results showed that both YM001 and HN016 can be colonized in the foregut, rectum, duodenum and hindgut, but their number and distribution were significantly different. The contents of YM001 and HN016 were the highest in all intestinal segments at 12 h and gradually decreased with time (Figures 1A,B). The relative reductions of YM001 and HN016 in different segments of the intestine were different. The reduction of YM001 was in the order of foregut> rectum> duodenum> hindgut, and hindgut> duodenum> rectum> foregut at 24 h−3d (Figure 1C) while that of HN016 was duodenum> foregut> hindgut> rectum (Figure 1D).

### 16S rRNA Gene Sequencing Analysis of Intestinal Microbiota in the Experimental Tilapia

The V3-V4 region of the 16S rRNA gene of each sample was sequenced on the Illumina HiSeq platform. A total of 2,891,942 original sequences and 2201874 effective reads were obtained from 36 samples. The total effective rate was 76.13%. The number of original sequences per sample was 63,842–92,065 and the effective read number was 48,541–70,400. The average length (AvgLen) was 405–426 nt (Table 2). To study the compositional diversity information of the microbiota, the effective sequences of all samples were clustered using the Uparse software into Operational Taxonomic Units (OTUs) according to 97% sequence similarity. A total of 964 OTUs were obtained from 36 tilapia intestinal samples with average OTUs of 339–1,623 per sample.

**Table 2:** Statistics the data of the samples sequencing.

**ID**	**Raw reads number**	**Effective reads number**	**Effective reads ratio (%)**	**AvgLen (bp)**
S1	67,202	51,024	75.93	407
Q1	87,705	63,545	72.45	415
H1	88,978	63,048	70.86	418
Z1	80,330	61,860	77.00	400
RS1	85,012	66,061	77.71	411
RQ1	69,790	53,911	77.25	399
RH1	77,753	60,507	77.82	411
RZ1	87,127	68,186	78.26	414
RS2	82,023	60,768	74.09	423
RQ2	77,184	56,278	72.91	425
RH2	90,328	67,106	74.29	425
RZ2	92,065	68,313	74.20	424
RS3	79,045	60,180	76.13	414
RQ3	80,394	62,708	78.00	408
RH3	76,219	59,523	78.09	413
RZ3	88,974	68,382	76.86	411
RS4	81,896	61,065	74.56	413
RQ4	84,224	67,639	80.31	412
RH4	75,255	58,985	78.38	410
RZ4	80,641	64,536	80.03	413
RS5	64,970	48,541	74.72	404
RQ5	80,847	63,449	78.48	407
RH5	75,724	60,644	80.09	407
RZ5	89,025	70,400	79.08	409
RS6	83,729	60,600	72.38	409
RQ6	63,842	50,847	79.65	403
RH6	81,714	59,235	72.49	407
RZ6	71,835	55,227	76.88	412
QS1	90,604	67,067	74.02	422
QQ1	86,118	65,332	75.86	416
QH1	69,187	52,006	75.17	415
QZ1	76,567	58,042	75.81	419
QS2	82,843	58,386	70.48	426
QQ2	82,196	63,483	77.23	412
QH2	77,184	60,891	78.89	425
QZ2	83,312	64,101	76.94	418

### Distribution of Microorganisms in Various Intestinal Segments of Tilapia Before Oral Administration of *S. agalactiae*

**Figure 2:**
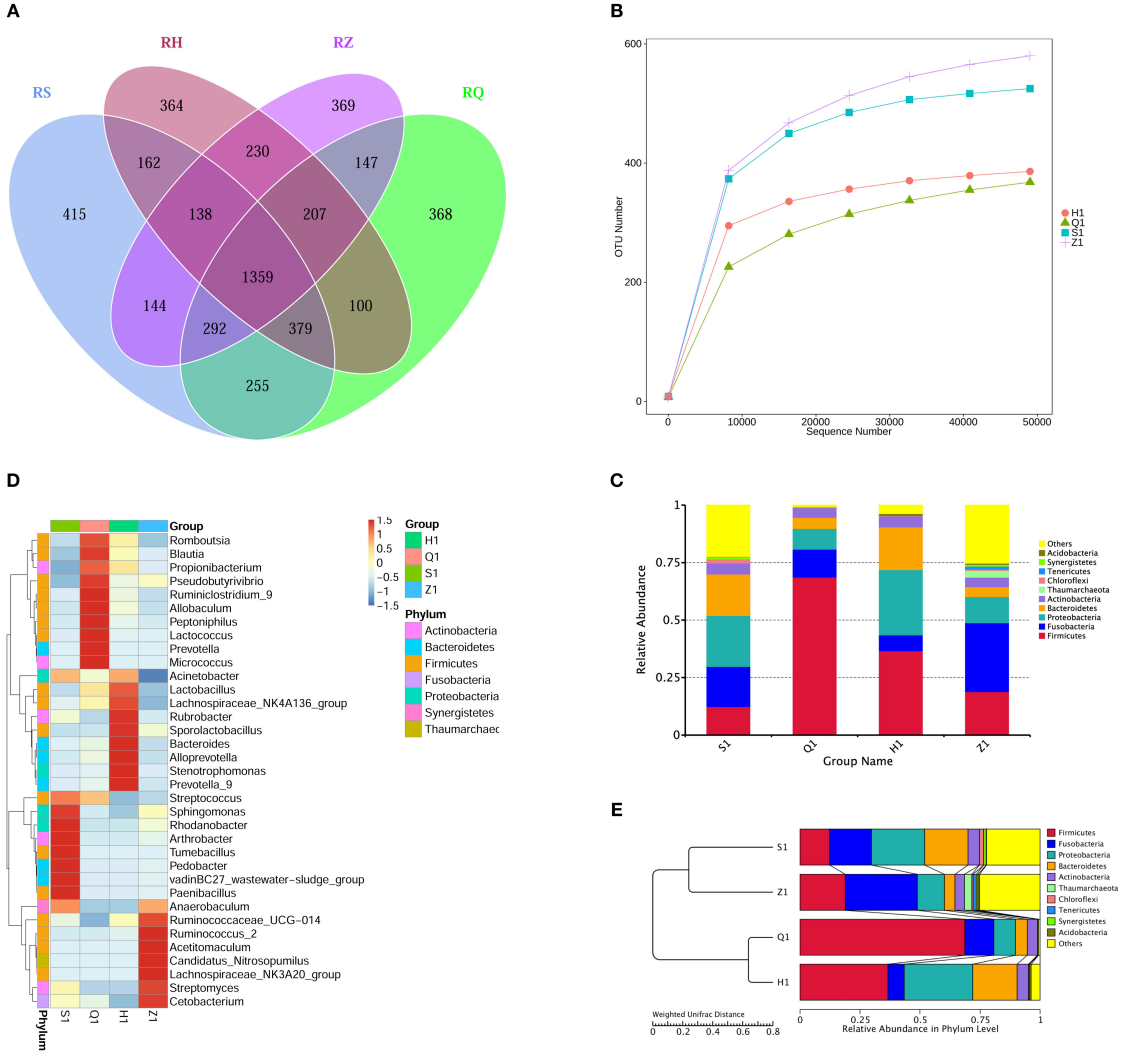
The intestinal flora in different segments of tilapia were different in structure and diversity. **(A)** Venn diagram (Petals figure) illustrating the Common or characteristic OTUs in duodenum (S), foregut (Q), midgut (Z), hindgut (H). **(B)** Rarefaction curves of OTUs illustrating the microbial diversity in duodenum (S), foregut (Q), midgut (Z), hindgut (H). When the curve flattens, it shows that the amount of sequencing data is gradually reasonable, and more data will produce only a small number of new species (OTUs). **(C)** Column diagram illustrating the relative abundance of Intestinal flora at phylum level in duodenum (S), foregut (Q), midgut (Z), hindgut (H). The horizontal axis is the sample name, and the vertical axis represents the relative abundance of a classification. Different colors correspond to different species at the same level. **(D)** Heat maps illustrating the relative abundance of Intestinal flora at genus level in duodenum (S), foregut (Q), midgut (Z), hindgut (H). The color and intensity of each square represent the value of relative abundance. **(E)** UPGMA clustering tree illustrating the similarity between samples of duodenum (S), foregut (Q), midgut (Z), hindgut (H).

Analysis of the Venn diagram of intestinal microbiota showed that 1,359 OTUs were shared in the intestine of normal tilapia, and the number of OTUs unique to the duodenum, foregut, hindgut, rectum was 415, 365, 364, and 469, respectively (Figure 2A).

At the phylum level, the dominant microbial floras in tilapia intestine are mainly firmicutes, fusobacterium, proteobacteria, bacteroidetes, actinobacteria, thaumarchaeota, chloroflexi, tenericutes, verrucomicrobia, synergistetes, and actidobacteria (Figure 2C). The dominant microbiota in tilapia duodenum were proteobacteria (22.14.38%), bacteroidetes (18.02%), fusobacterium (17.41%), firmicutes (12.39%), actinobacteria (4.77%), chloroflexi (1.79%), and Others (23.37%); The dominant microbiota in tilapia foregut were Firmicutes (68.67%), fusobacterium (12.12%), proteobacteria (8.99%), bacteroidetes (4.86%), actinobacteria (4.23%), and others (1.12%); The dominant microbiota in tilapia hindgut were Firmicutes (36.66%), proteobacteria (28.41%), bacteroidetes (18.56%), fusobacterium (6.84%), actinobacteria (4.68%), Chloroflexi (1.79%), Others (23.37%); The dominant microbiota in tilapia rectum were fusobacterium (29.96%), firmicutes (18.86%), proteobacteria (11.35%), bacteroidetes (4.32%), actinobacteria (4.08%), thaumarchaeota (2.99%), tenericutes (1.29%), and others (27.14%).

At genus level, different positions of tilapia intestine also have different dominant bacterial structures (Figure 2D). The dominant microbiota in tilapia duodenum were *streptococcus, sphingomonas, rhodanobacter, arthrobacter, tumebacillus, pedobacter, vadinBC27_wastewater-sludge_group*; *paenibacillus* and *anaerobaculum*; the dominant microbiota in tilapia foregut were *romboutsia, blautia, propionibacterium, pseudobutyrivibrio, ruminiclostrid_9, allobaculum, peptoniphilus, lactococcus, prevotella* and *micrococcus*; the dominant microbiota in tilapia hindgut were *Acinetobacter, Lactobacillus, Lachnospiraceae_NK4A136_group, Rubrobacter, Sporolactobacillus, Bacteroides Alloprevotella, Stenotrophomonas and Prevotella_9;* and the dominant microbiota in tilapia rectum were *Anaerobaculum, Ruminococcaceae_UCG-014, Ruminococcus_2, Acetitomaculum, Candidatus_Nitrosopumilus, Lachnospiraceae_NK3A20_group, Streptomyces, Cetobacterium*.

The clustering tree analysis showed that the microbiota in tilapia foregut and hindgut were more similar than that in the duodenum and rectum (Figure 2E). The dilution curve can directly reflect the rationality of the sequencing data and indirectly reflect the abundance of species in the sample. Our results in the dilution curves showed that the diversity of microbes in different gut segments of tilapia was different, showing an order of rectum> duodenum> hindgut> foregut in species richness (Figure 2B).

### Effect of YM001 on Intestinal Microbiota in Tilapia

**Figure 3:**
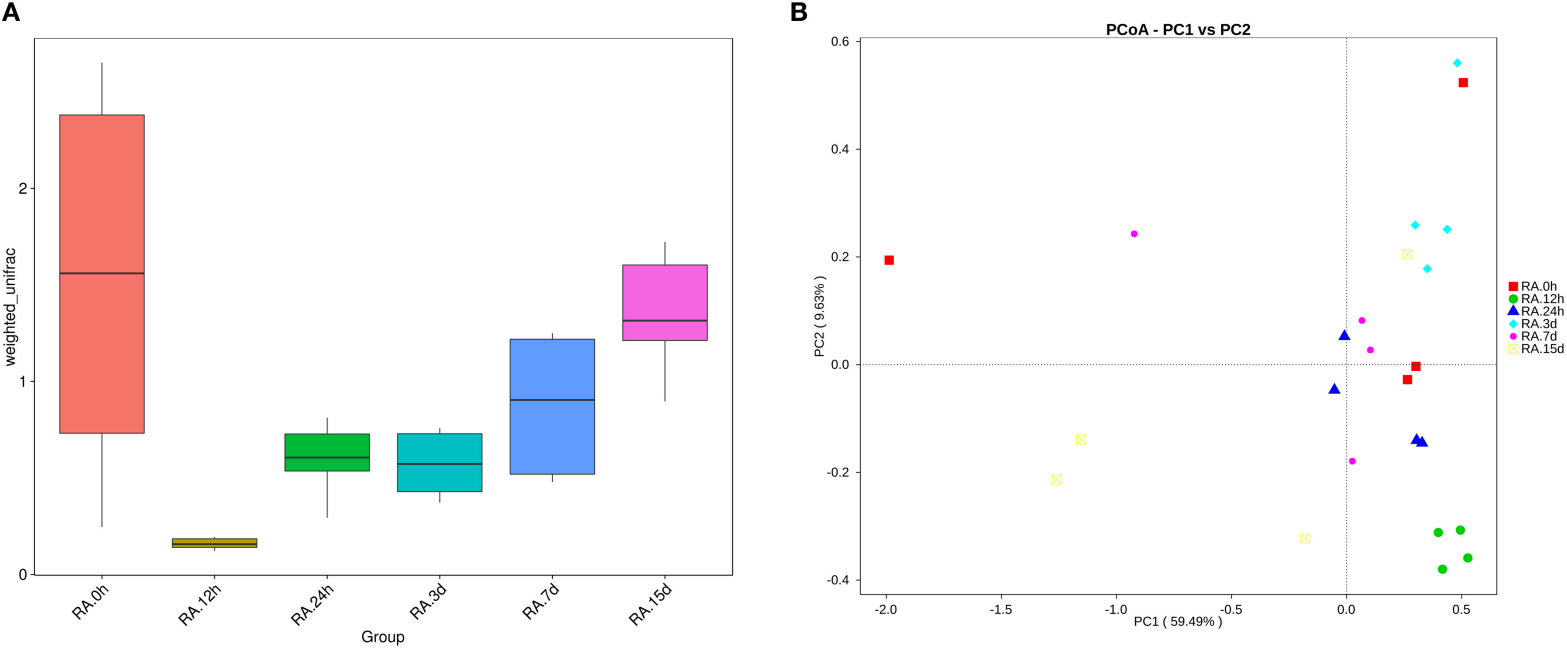
YM001 alters the diversity of intestinal flora of tilapia at different observing times. **(A)** A box chart based on Weighted Unifrac Beta diversity. **(B)** Principle coordinate analysis (PCoA) Based on Weighted Unifrac distance. The samples at 0 h, 12 h, 24 h, 3 d, 7d, 15d were represented as color icons with red square, green dot, blue triangle, turquoise square, pink dot, orange box.

**Figure 4:**
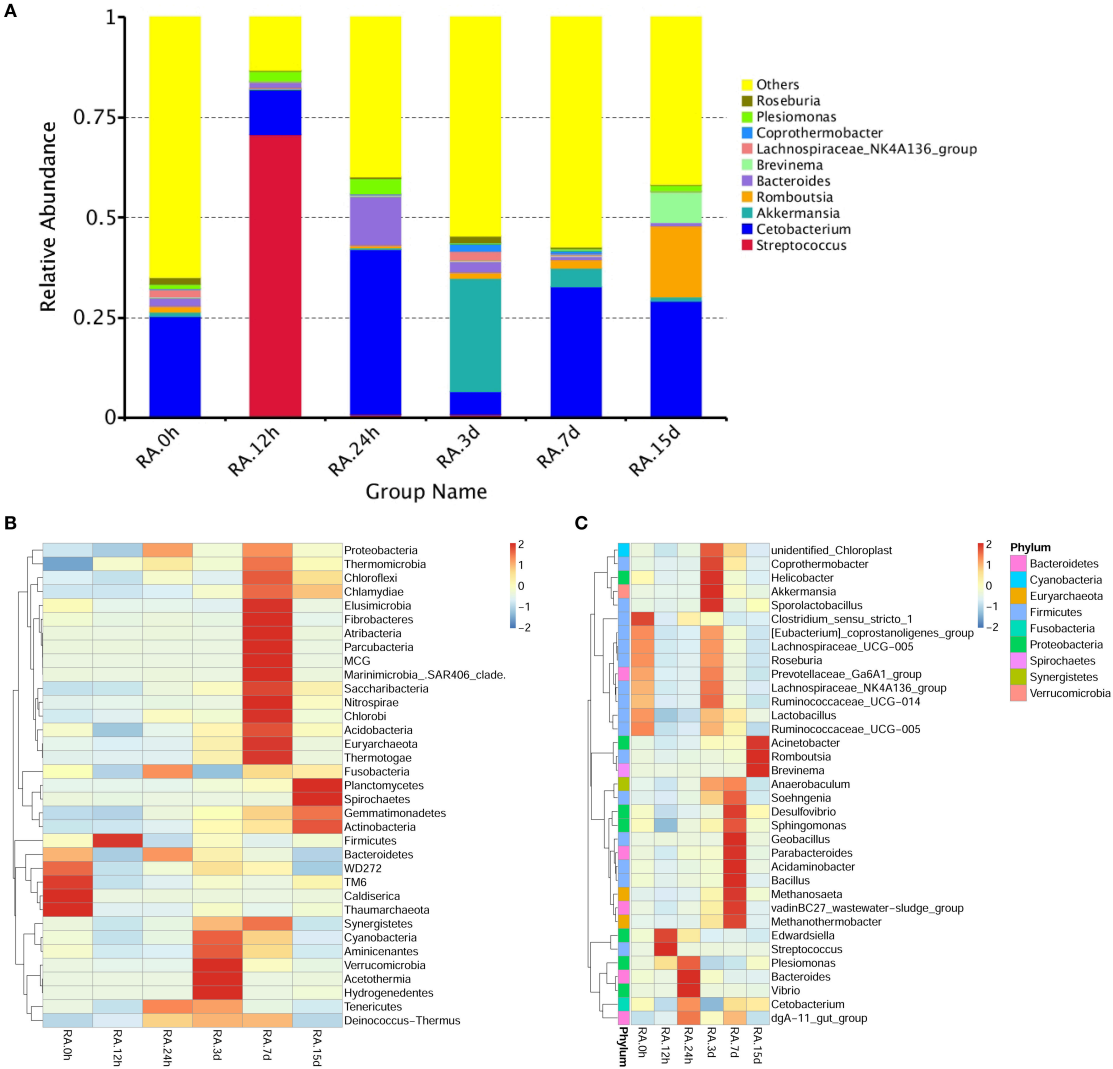
YM001 alters the intestinal flora composition at different observing times. **(A)** The relative abundances of the top 10 bacteria at the genus level in YM001 groups. **(B)** The alteration of intestinal bacterial patterns at the genus level. The heat map is color-based on row Z-scores. The highest and lowest bacterial levels are showed in red and blue, respectively. **(C)** The alteration of intestinal bacterial patterns at the phylum level. The heat map is color-based on row Z-scores. The highest and lowest bacterial levels are showed in red and blue, respectively.

β diversity analysis of intestinal samples of tilapia found that the diversity of gut microbiota lowered after oral administration of YM001, reaching the lowest at 12 h and gradually recovered afterward to the normal level at 15 d (Figure 3). Furthermore, YM001 significantly changed the composition of microbiota in tilapia intestine, and the relative proportion of dominant bacteria fluctuated at different time (Figure 4A). At genus level, the top 10 intestinal microbe were Streptococcus, *Cetobacterium, Akkermansia, Romboutsia, Brevinema, Lachnospiraceae_NK4A136-group, Coprothermobactter, Plesiomonas*, and *Roseburia* after the YM001 administration. The abundance of Streptococcus in the intestine reached the highest level (70.8%) at 12 h after YM001 administration, then dramatically decreased at 24 h after YM001 administration, and almost restored to the normal level at 5 d and 7 d after YM001 administration (Table 3). The trend of changes in *Streptococcus* abundance is consistent with that measured by fluorescence quantitative PCR (Figure 1). *Cetobacterium* accounted for approximately 25% of the total bacteria before oral administration of YM001 and showed a significant decrease at 12 h after administration. After that, the abundance of *Cetobacterium* rebounded to 25% at 24 h, decreased again at 3 d after administration and almost returned to the original level at 7 and 15 d after administration (Table 3). The abundance of *Bacteroides* was about 2.2% before oral administration of YM001 but increased to 12.4% at 24 h after administration and then gradually decreased to the original level later (Table 3). *Akkermansia* accounted for about 1% of the total bacteria before oral administration of YM001, but the abundance rose to 28.3% at 3 d after oral administration of YM001 and then gradually decreased at 5 and 7 d (Table 3). *Romboutsia* accounted for about 1.5% of the total bacteria before oral administration of YM001, but the abundance reduced to 0.8% at 12 h after oral administration of YM001 and then gradually increased to 17.7% at 15 d (Table 3). The content of *Brevinema* increased to 7% at 15 d after oral administration of YM001, and was less than 1% at all other times (Table 3).

**Table 3:** The composition of the top ten intestinal flora in YM001 group.

**Taxonomy**	**Strep**	**Cetob**	**Akker**	**Romb**	**Bacte**	**Brevi**	**Lachn**	**Copro**	**Plesi**	**Roseb**	**Others**
RA.0h	0.005012	0.247655	0.01045	0.015298	0.022154	0.001102	0.019525	0.001347	0.011202	0.017022	0.649234
RA.12h	0.708163	0.110283	0.002242	0.002841	0.014807	0.000169	0.001974	0.00042	0.024902	0.001407	0.132791
RA.24h	0.008764	0.411216	0.003523	0.006643	0.124136	0.000976	0.003	0.000807	0.039513	0.002274	0.399147
RA.3d	0.008623	0.056982	0.283192	0.014076	0.028616	0.000774	0.024422	0.018505	0.001767	0.016662	0.54638
RA.7d	0.004887	0.322307	0.046462	0.020419	0.009822	0.000862	0.005208	0.007788	0.004058	0.004483	0.573704
RA.15d	0.001756	0.290315	0.009332	0.177731	0.00895	0.076065	0.002476	0.00018	0.013984	0.001189	0.418023

*Strep, Streptococcusl; Cetob, Cetobacterium; Akker, Akkermansia; Romb, Romboutsia; Bacte, Bacteroides; Brevi, Brevinema; Lachn, Lachnospiraceae_NK4A136_group; Copro, Coprothermobacter; Plesi, Plesiomonas; Roseb, Roseburia*.

The contents of *Lachnospiraceae_NK4A136-group, Coprothermobactter, Plesiomonas*, and *Roseburia* in the intestine also fluctuated but to less extent compared with the other genera. Before oral administration of YM001, the content of *Lachnospiraceae_NK4A136-group* was 7% in duodenum and <1% in other segments, after oral administration of YM001, its content showed a decreasing trend (Table 3). The content of Presiomonas was approximately 0.4% in total intestinal bacteria in the case of normal, but after oral administration of YM001, its content rose to 2.4% at 12 h and 3.9% at 24 h, and then gradually reduced to 1.3% at 15 d, close to the initial level (Table 3). Before oral administration of YM001, the content of *Coprothermobactter* in each gut segment was <0.1%. However, at 3 d after administration of YM001, its content in the foregut increased to 7% and then returned to the normal level at 7 and 15 d after administration of YM001 (Table 3). Before oral administration of YM001, the content of *Roseburia* in each gut segment was < 0.1% except that of 6.3% in duodenum. After administration, its content in duodenum gradually decreased, reaching to the normal level at 7 and 15 d after administration of YM001 (Table 3).

In addition to the above-mentioned predominant bacteria, oral administration of YM001 also caused changes in other non-predominant gut bacteria. The heat maps show the changes in the highest 35 gut bacteria at both phylum and genus levels (Figures 4B,C).

### Differences of Gut Microbial Changes in Tilapia Subjected to Oral Administration of YM001 and HN016

**Figure 5:**
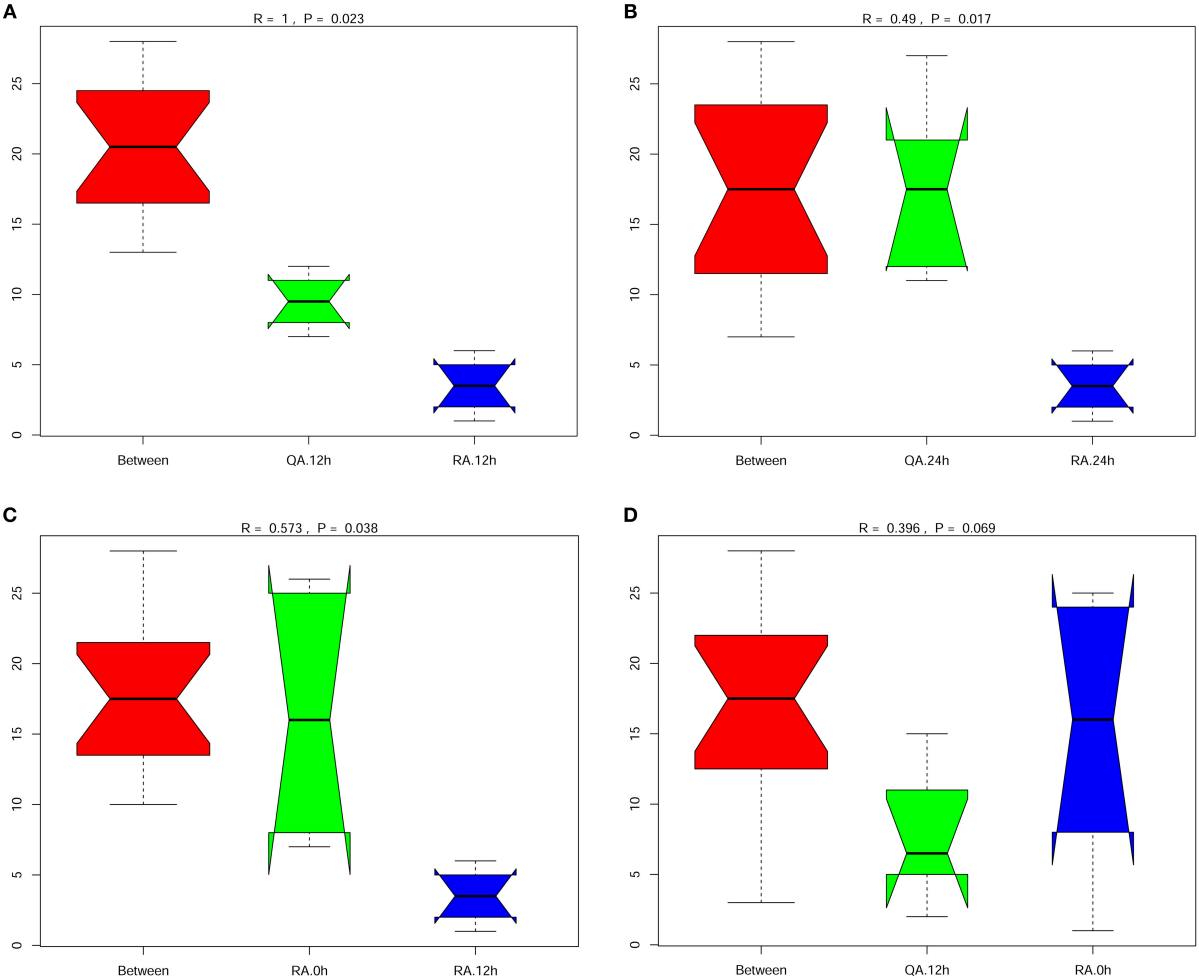
Significant difference between the group oral with YM001 and the group fed with HN016. **(A)** The differences between group R (YM001) and group Q (HN016) at 12 h by Anosim analysis. **(B)** The differences between group R (YM001) and group Q (HN016) at 24 h. **(C)** The differences of group R (YM001) between 12 h and 24 h. **(D)** The differences of group Q (HN016) between 12 and 24 h. *R*-value is between (−1, 1) and *R*-value is >0, indicating significant difference between groups. *R*-value is < 0, indicating that the difference in the group is greater than that between groups. The credibility of statistical analysis is expressed in *p*-value, and *P* < 0.05 indicates that the statistics are significant.

Anosim analysis is a non-parametric test used to examine whether the differences between groups are significantly greater than the differences within groups. Comparison of intestinal microbiota composition between any two group, YM001 and HN016 or normal control group (0 h), showed that all the R-values of Anosim analysis were >0 (Figures 5A–D), indicating that the composition of intestinal microbiota between any two group was significantly different and the difference between the groups were greater than that within the group.

**Figure 6:**
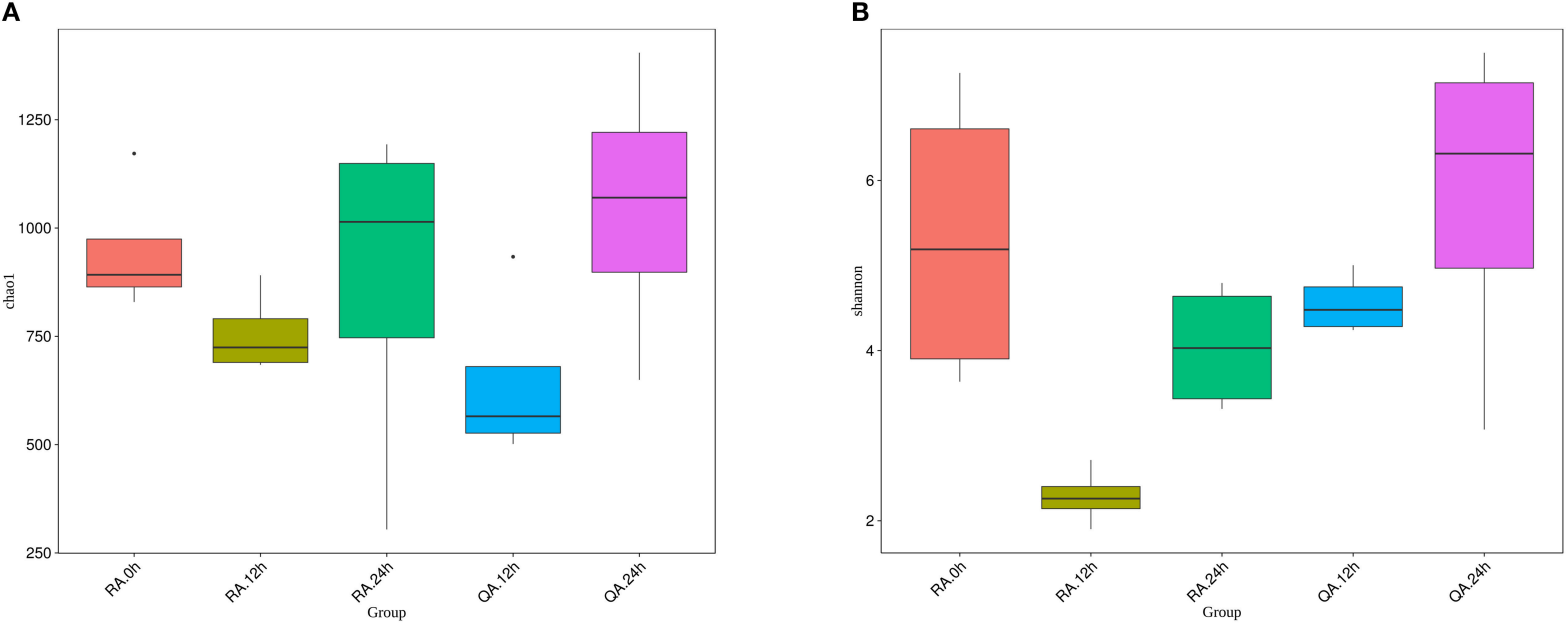
The diversity of intestinal flora of tilapia, which fed with YM001 or HN016, changed differently. **(A)** Diversity of bacterial species as indicated by Chao1 rarefaction measure. The larger the number of OTU in the sample is, the larger the number of species in the sample. **(B)** Shannon index. The richness and evenness of the species composition in the sample were evaluated. The larger the value was, the more abundant the species was, and the more homogeneous the species were. Evenness based on species-level operational taxonomic units (OTUs). Error bars indicate SD,^*^ indicates significant association (0.005 < *P* < 0.05).

To determine whether two different *S. agalactiae* strain altered the composition of the gut microbiota in a similar pattern, we compared the diversity and richness indices of intestinal microbiota of tilapia at different periods after administration of YM001 and HN016. The results showed that both Chao1 index and Shannon index dropped at 12 h after administration of YM001 or HN016, then increased again at 24 h. Especially, the Chao1 index of YM001 or HN016 groups at 24 h was higher than that of the control groups. Except the Chao1 index at 12 h, the Chao1 index and the Shannon index of YM001 groups at all other time points were lower than those of HN016 groups (Figures 6A,B). These data indicated that the richness of the intestinal microbiota of tilapia at 12 h after administration of YM001 or HN016 were attenuated, especially in tilapia after 12 h after oral administration of YM001.

**Figure 7:**
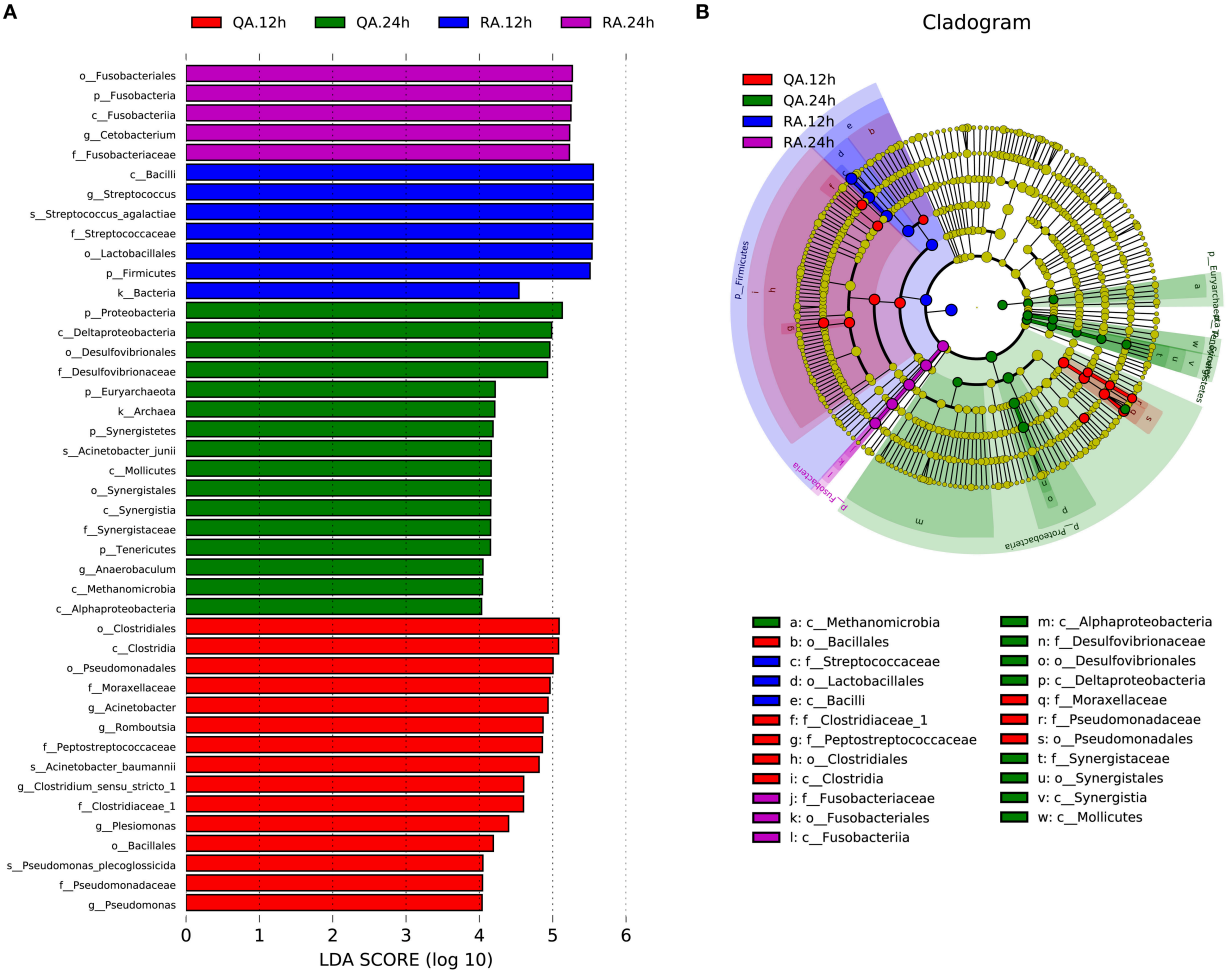
YM001 and HN016 alter the intestinal flora composition differently. **(A)** The taxa whose abundance differed between the RA samples (blue and violet bars) and QA samples (red and green bars). The cutoff value of ≥4.0 used for the linear discriminant analysis (LDA) is shown. **(B)** Taxonomic cladogram obtained from LEfSe sequence analysis (relative bundance ≥ 0.5%). Biomarker taxa are highlighted by colored circles and shaded areas (RA samples are shown in blue or violet and QA are shown in red or green). Each circles diameter reflects the abundance of that taxa in the community.

LDA effect size (LEfSe) analysis is used to identify biomarkers or the species that have statistically significant differences between two groups. Comparing with other groups, the following biomarkers, including *k__Bacteria, p__Firmicutes, c__Bacilli, o__Lactobacillales, f__Streptococcaceae, g__Streptococcus and s__Streptococcus_agalactiae*, of YM001 group at 12 h were most different (Figures 7A,B). On other hand, the following biomarkers, including *p__Fusobacteria, c__Fusobacteriia, o__Fusobacteriales, f__Fusobacteriaceae, g__Cetobacterium*, of YM001 group at 24 h were most different compared with other groups. In addition, the biomarkers *c__Clostridia, o__Clostridiales, o__Pseudomonadales, o__Bacillales, f__Clostridiaceae_1, f__Pseudomonadaceae, f__Peptostreptococcaceae, f__Moraxellaceae, g__Pseudomonas, g__Plesiomonas, g__Romboutsia, g__Acinetobacter, g__Clostridium_sensu_stricto,s__Acinetobacter_baumannii, s__Pseudomonas_plecoglossicida* were significantly different in the intestine of tilapia at 12 h after administration of subjected to virulent *S. agalactiae* strain HN016 compared with other groups; and the biomarkers *k__Archaea, p__Tenericutes, p__Synergistetes, p__Euryarchaeota, p__Proteobacteria, c__Alphaproteobacteria, c__Methanomicrobia, c__Mollicutes, c__Synergistia c__Deltaproteobacteria, o__Synergistales, o__Desulfovibrionales, f__Desulfovibrionaceae, f__Synergistaceae, g__Anaerobaculum, and s__Acinetobacter_junii* were significantly different in the intestine of tilapia at 24 h after administration of subjected to HN016 compared with other groups (Figures 7A,B).

## Discussion

For oral attenuated vaccines, safety and efficacy are critical. In this study, each tilapia was given 10^9^ YM001 or 10^9^ HN016 via oral gavage, respectively. Compared with 100% mortality of fish given HN016 within 3 d, the mortality of fish given YM001 was 0% within 15 d, indicating that YM001 has higher safety. The decrease of virulence of YM001 was related to the change of its genetic structure. Our previous data have shown that compared to HN016, YM001 lost two 5832bp, and 11,116 bp DNA fragments and possesses more than 40 genes with deletions or point mutations (Wang et al., 2015).

Live vaccines administrated via gavage in the intestine could stimulate immunity through two routes. On the one hand, live bacteria will colonize on the mucosa of intestine, stimulating mucosal immunity. On the other hand, these bacteria will break through the intestinal barrier and enter other immune organs, stimulating systemic immunity. Therefore, the number of colonization in the intestine, retention time, and intestinal barrier breakthrough efficiency of YM001 are related to the immune efficacy of the YM001 oral vaccine. In this study, real-time quantitative PCR was used to detect the retention of YM001 and HN016 in the intestine of tilapia. It was found that the content of both was the highest at 12 h after administration and then greatly reduced to almost undetectable at 3d (HN016) or 7d (YM001) after administration (Figure 1A). The above results show that the intestinal environment of tilapia is not suitable for long-term colonization of YM001. The intestine as well as its intrinsic microbiota has a strong repulsive effect on YM001.

The symbiotic bacteria colonize in the intestinal mucosa of fish to form a bacterial plexus, which acts as a barrier against the colonization and proliferation of pathogens and to a certain extent protects the host against pathogens (Gómez and Balcazar, 2008; Kelly, 2010). Our previous studies showed that YM001 could be detected in the brain, liver, spleen, and kidney of tilapia at 6 h after administration of YIM001, and the levels of YM001 in the tissues gradually decreased after 24 h of YM001 administration (Li et al., 2015), indicating that although YM001 is repulsed by the intestine or intrinsic microbiota of tilapia, it can still quickly cross the intestinal barrier to reach other parts of the body. The content of YM001 in the intestine and other organs began to decrease simultaneously at 24 h after oral administration, indicating that the defense responses of intestine in tilapia are highly synchronized with the internal defense responses of the body. The responses of tilapia intestine to YM001 and HN016 may be different. Comparison of the contents of YM001 and HN016 in various segments of the intestine showed that there was still a large amount of YM001 in tilapia intestine at 3 d after oral administration, but HN016 was almost undetectable, indicating that YM001 was likely to colonize on the intestine much longer that than HN016 (Figures 1A,B). In addition, comparison of the content of YM001 and HN016 in each segment of the intestine also showed that the concentration of YM001 was higher than that of HN016 at each time point except that the concentration of YM001 was lower than that of HN016 in foregut at 24 h after administration (Figures 1A–D). There may be two reasons for this situation. One is that the virulent strain HN016 enters the intestinal barrier faster than the attenuated strain YM001, resulting in a lower content of HN016 in the intestine than that of the attenuated strain YM001. The other one is because HN016 could produce toxins, resulting in strong intestine responses, which accelerate the discharge of HN016, and eventually result in lower content in the intestine than that of the attenuated strain YM001. Regardless of the situation, the longer colonization time of the weak strain YM001 in the intestine is obviously beneficial to stimulate the body to produce long-lasting immunity.

To analyze the influence of YM001 on intestinal microbiota of tilapia, it is necessary to understand the structure of the intestinal microbiota of healthy tilapia. 16S RNA sequencing analysis showed that *proteobacteria, firmicutes, fusobacterium, bacteroidetes, verrucomicrobia, spirochaetes, Euryarchaeota, actinobacteria, synergistetes*, and *tenericutes* are the top abundant bacteria genera in the intestine of healthy tilapia (Figure 2C). Swapnil Sopan Gaikwad et al. used 16S rRNA gene sequencing on the Illumina Miseq platform and found that *Fusobacteria* and *Proteobacteria* are the dominated genera in the digestive tract of tilapia collected from rivers while *Actinobacteria, Cyanobacteria, Planctomyctes*, and *Proteobacteria* are the dominant genera in in the digestive tract of tilapia collected from lakes (Gaikwad et al., 2017). Our results are similar to those of Swapnil Sopan Gaikwad et al., but there are also some bacterial species that are inconsistent with their research, such as *Verrucomicrobia, Spirochaetes, Euryarchaeota, Synergistetes*, and *Tenericutes*. These discrepancies may be related to the experimental fish species, different living environments and diet, and may also be related to different sequencing methods and sequencing depths. Microbial colonies are widely distributed in the digestive tract and are found in almost every segment of the digestive tract. At the same time, unique colonies are formed in various segments of the digestive tract. To our best knowledge, there are no reports about the bacterial structures in different segments of the digestive tract in tilapia. We found that there are significant differences in the microbial composition at different segments of the digestive tract of tilapia (Figures 2A,B). The differences in microbiota at different parts of segments of the digestive tract are closely related to their physiological characteristics.

The diversity and abundance of intestinal microbiota in animals may change due to factors such as age, dietary changes, medication, and infection (Xia et al., 2014). One instance is that canine distemper virus infection distorted the intestinal microbiota composition by reducing the prevalence of the dominant genera, Escherichia and Clostridium, and increasing microbial diversity. In this study, we performed high-throughput 16S RNA gene sequencing of the intestinal microbiota of tilapia after oral administration of *S. agalactiae* YM001. Through β diversity analysis, it was found that early oral YM001 immunization could decrease intestinal microbiota diversity in tilapia (Figure 3A). Changes in intestinal microbiota may have an impact on the health of the host. Previous studies have shown that changes in intestinal microbiota may have an impact on the health of the host. For human, the decrease in bacterial richness and diversity is consistent with other disease states that have alterations in the intestinal microbiota such as inflammatory bowel disease and diabetes (Jandhyala et al., 2017; Nishino et al., 2018). Despite this, administration of YM001 only temporarily reduced the diversity of intestinal microbiota in tilapia (Figures 3A,B) and changes in intestinal microbial diversity in tilapia appear to be closely related to YM001 concentration in the intestine. By analyzing the relationship between the retention of oral streptococcus and the diversity of intestinal microbiota, we found that when the concentration of YM001 was highest, the diversity of intestinal microbiota in tilapia was the lowest, and with the concentration of YM001 decreasing, the diversity was gradually recovered (Figures 3A,B). Oral administration of virulent strain HN016 also decreased the intestinal microbiota diversity of tilapia, but as the concentration of HN016 decreasing, the intestinal microbiota diversity also recovered or even exceeded the normal level (Figures 6A,B). More and more scholars started to explore the effects of oral vaccines on intestinal microbiota in recent years. It has been reported that oral administration of live-attenuated typhoid vaccine Ty21a could considerably affect inter- and intra-individual variability, yet not discernibly perturb the bacterial assemblage related to vaccine administration (Eloe-Fadrosh et al., 2013). Oral administration of different *S. Typhimurium* vaccine strains can differentially influence the presence, but not the relative abundance of microbiota in the ceca (Park et al., 2017). Similar studies have also been reported in aquatic animals. When bath-immunizing grass carp using recombinant *Aeromonas hydrophila* vaccine (Aera), there was no significant difference in the abundance of genera *Acinetobacter, Cetobacterium*, and *Gemmobacter* compared to the Control (Liu et al., 2015). All the above examples indicate that oral vaccines can affect the composition of the intestinal microbiota to varying degrees.

Intestinal microbiota imbalance could cause host health problems, which may be related to reduced levels of beneficial bacteria and increased levels of pathogenic bacteria. By analyzing the microbiota changes in the different gut segments, it was found that at the genus level, the contents of *Streptococcus, Cetobacterium, Akkermansia, Romboutsia, Bacteroides, Brevinema* and *Lachnospiraceae_NK4A136* are changed the most after administration of YM001 (Figure 4A). Ecept the *Streptococcus* which are directly related to YM001, the function of the other above bacteria are as follow. *Cetobacterium* is bile resistant and could produce acetic acid as the major end product of metabolism of peptides and carbohydrates (Finegold et al., 2003). *A. muciniphila* is a mucin-degrading bacterium and inversely associated with obesity, diabetes, inflammation, and metabolic disorders (Everard et al., 2013; Caesar et al., 2015). *Romboutsia* can utilize different relatively simple carbohydrates and synthesize limited amino acids and vitamins (Gerritsen et al., 2017). *Bacteroides* play a fundamental role in processing complex molecules to simpler ones in the host intestine (Wexler, 2007). *Bacteroides* species also benefit their host by excluding potential pathogens from colonizing the gut (Wexler, 2007). Some species (*B. fragilis*, for example) are opportunistic human pathogens (Wexler, 2007). *Brevinema* belongs to spirochaetes, which have been extensively studied in termite guts and have been found to be involved in the breakdown of lignocellulose and nitrogen fixation. *Lachnospiraceae* are a family of bacteria in the order of Clostridiales. Members of Lachnospiraceae family could produce butyric acid and are important for host epithelial cell growth (Meehan and Beiko, 2014). Among these bacteria, the change of *Cetobacterium* is particularly noteworthy. As the predominant bacteria in the intestine of tilapia, *Cetobacterium* accounted for 24.87% of the total intestinal bacteria, and its content increased to 41.1% at 24 h after oral administration of YM001, and gradually returned to 32.2 and 29% at 7 d and 15 d after oral administration of YM001. Streptococcus accounted for 0.5% of the total intestinal bacteria in healthy tilapia. At 12 h after oral administration of YM001, its content increased to up to 70.8% and then gradually returned to 0.8% at 24 h after oral administration of YM001. The results showed that oral YM001 changed the composition of intestinal microbiota in a short time; but with the decrease of YM001 content, the content of the predominant bacteria was gradually recovered.

The effects of attenuated strain YM001 and virulent strain HN016 on tilapia intestinal microbiota were very different. The differences in gut bacteria between these two groups were greater than the differenced within each group (Figure 5). Although oral administration of HN016 and YM001 both decreased the intestinal flora diversity, the effect of YM001 was more significant (Figure 6). The difference in intestinal flora caused by virulent strains and attenuated strains may be related to the secretion or metabolites of different strains. The specific underlying mechanisms need to be further explored.

In conclusion, after oral administration, YM001 could colonize in the digestive tract of tilapia for a certain period of time, and alter the structure and diversity of the intestinal microbiota. However, these changes do not have fatal consequences. Tilapia intestinal microbiota has a repulsive effect against YM001. With the content of YM001 in the digestive decreasing, the structure and diversity of intestinal microbiota gradually recovered.

## Author Contributions

ML, LL, and TH contributed equally to this work. LL, TH, YL, AL, and FC performed experiments and analyzed data. ML and CM provided software and bioinformatics expertise. MC and ML designed the experiments, analyzed data, and wrote the manuscript.

### Conflict of Interest Statement

The authors declare that the research was conducted in the absence of any commercial or financial relationships that could be construed as a potential conflict of interest.
